# Eculizumab for ATCR-associated *de novo* thrombotic microangiopathy in a Chinese kidney transplant recipient with type 2 diabetes: a case report

**DOI:** 10.3389/fmed.2026.1872856

**Published:** 2026-08-12

**Authors:** Shunye Su, Yan Zhu, Guiyan Han, Zhentao Zhang, Liqin Liang, Yuan Gao

**Affiliations:** 1 Department of Urology, Weifang People’s Hospital, Shandong Second Medical University, Weifang, Shandong, China; 2 Department of Pathology, Weifang People’s Hospital, Shandong Second Medical University, Weifang, Shandong, China; 3 School of Clinical Medicine, Shandong Second Medical University, Weifang, Shandong, China; 4 Department of Hospital Outpatient, Weifang People’s Hospital, Shandong Second Medical University, Weifang, Shandong, China

**Keywords:** ATCR, *de novo*, eculizumab, kidney transplantation, thrombotic microangiopathy

## Abstract

**Objective:**

This study aimed to explore the efficacy of eculizumab in the treatment of newly developed thrombotic microangiopathy (TMA) associated with acute T-cell-mediated rejection (ATCR) in patients with type 2 diabetes after kidney transplantation.

**Methods:**

New-onset thrombotic microangiopathy (TMA) after kidney transplantation is a relatively rare but serious complication that is clinically challenging to manage and requires timely intervention to prevent graft dysfunction or early graft loss. Currently, there is no consensus in clinical practice regarding the diagnosis, optimal treatment strategies, or preventive measures for post-transplant new-onset TMA. This article provides a retrospective analysis of the first case in China successfully treated with eculizumab for *de novo* TMA occurring in the context of acute T-cell-mediated rejection (ATCR), type 2 diabetes, and post-transplant immunosuppression.

**Patient:**

The patient was a 59-year-old male who underwent cadaveric donor kidney transplantation on July 31, 2024. The surgery was successful, and postoperative recovery was good. He was followed up in the outpatient department every week. On September 15, 2024, the patient experienced acute pain and swelling in the right lower abdomen, and his 24-h urine output decreased to 1,200 mL. After admission, biopsy of the transplanted kidney was performed, and severe thrombotic microangiopathy (TMA) was diagnosed. After pathological diagnosis, eculizumab was administered for 3 months (900 mg/time in the first four weeks, once a week; 1,200 mg/time from the fifth week to the 12th week, once a week).

**Results:**

After eculizumab treatment, the patient’s clinical symptoms improved significantly. Laboratory indicators, such as platelet count, lactate dehydrogenase (LDH), and serum creatinine (SCr) gradually returned to normal. During the 9-month follow-up after the last dose, the SCr level remained stable at 180 μmol/L, with no recurrence of thrombotic microangiopathy (TMA) and no signs of proteinuria were observed.

**Conclusion:**

Eculizumab is effective in treating new-onset ATCR-related TMA in patients with type 2 diabetes after kidney transplantation, and treatment should be initiated immediately after diagnosis.

## 1 Background

Transplantation-associated thrombotic microangiopathy (TA-TMA), resulting from over activation and dysregulation of the alternative complement pathway, represents a severe life-threatening complication following kidney transplantation (1). This condition most frequently manifests within the first six postoperative months (2). The complement system in TA-TMA may be activated by multiple factors, including autoimmune diseases, antibody-mediated rejection (ABMR), immunosuppressive agents, infections, ischemia–reperfusion injury, malignancies, antiphospholipid antibody syndrome, systemic sclerosis, C3 glomerulopathy, anti-VEGF medications, and complement-related gene mutations, all of which contribute to endothelial injury pathogenesis.

TA-TMA is a multifactorial disorder characterized by thrombocytopenia, hemolytic anemia, reduced haptoglobin levels, elevated LDH, and multiorgan dysfunction, particularly acute kidney injury (3). Reported incidence rates range from 0.8 to 15%, with graft loss occurring in 33–50% of cases (4). While the annual incidence is approximately 0.56%, the three-year mortality rate exceeds 50% in confirmed cases (5). Diagnostic criteria require fulfilment of TMA parameters after excluding thrombotic thrombocytopenic purpura (TTP), Shiga toxin-producing *Escherichia coli*-associated hemolytic uremic syndrome (STEC-HUS), and secondary TMA forms (including malignancy, connective tissue disorders, pregnancy, and causative medications). Genetic analyses revealed pathogenic variants in complement genes (CFH, CFI, C9, and C3) among most living donor kidney transplant recipients, with CFH mutations demonstrating particularly high recurrence rates (6). Patients carrying CFH mutations or CFH/CFHR1 hybrid genes exhibit an elevated risk of recurrence despite PE prophylaxis. TA-TMA classification encompasses immunosuppressant-induced TMA, ABMR-associated TMA, infection-related TMA, and *de novo* or recurrent TMA. Histopathological hallmarks include arteriolar and glomerular vascular damage, featuring intraluminal thrombosis, fibrinoid necrosis, marked arteriolar wall thickening, glomerular basement membrane duplication, and mesangiolysis. Current management lacks standardization, with high mortality persisting despite PE and IVIG therapies.

Eculizumab, a humanized anti-C5 monoclonal antibody that inhibits C5b-9 membrane attack complex formation, has demonstrated efficacy in both native kidney and transplant-associated TMA.

## 2 Case presentation

A 59-year-old male patient (blood type AB) with idiopathic end-stage renal disease (ESRD) for over two years—genetic testing negative, no family history of kidney disease, and normal renal biopsy results. The patient had a 15-year history of hypertension and 5-year history of type 2 diabetes mellitus. To establish vascular access, an arteriovenous fistula (AVF) catheter was inserted into the left forearm, and regular hemodialysis therapy was initiated in May 2023. Over 14 months, the patient underwent high-efficiency hemodialysis three times weekly, with preoperative investigations showing negative complement-dependent cytotoxicity (CDC) and absence of donor-specific anti-HLA antibodies (DSA). He was subsequently admitted from July 31 to August 10, 2024, for ABO-compatible deceased donor kidney transplantation from a donation after brain death (DBD) donor with four HLA mismatches. The perioperative immunosuppressive regimen comprised cyclosporine A, mycophenolic acid sodium, prednisone, methylprednisolone, and anti-human T cell immunoglobulin (ATGF). Graft reperfusion was satisfactory, with immediate postoperative Doppler demonstrating good perfusion and normalized renal function (blood urea nitrogen 7.1 mmol/L, serum creatinine, 97 μmol/L; urine output, 120 mL/h). The cyclosporine trough levels remained within the therapeutic range (94–257.9 ng/mL) throughout the perioperative period. The patient was discharged on postoperative day 7 with a scheduled weekly outpatient follow-up.

**Figure 1:**
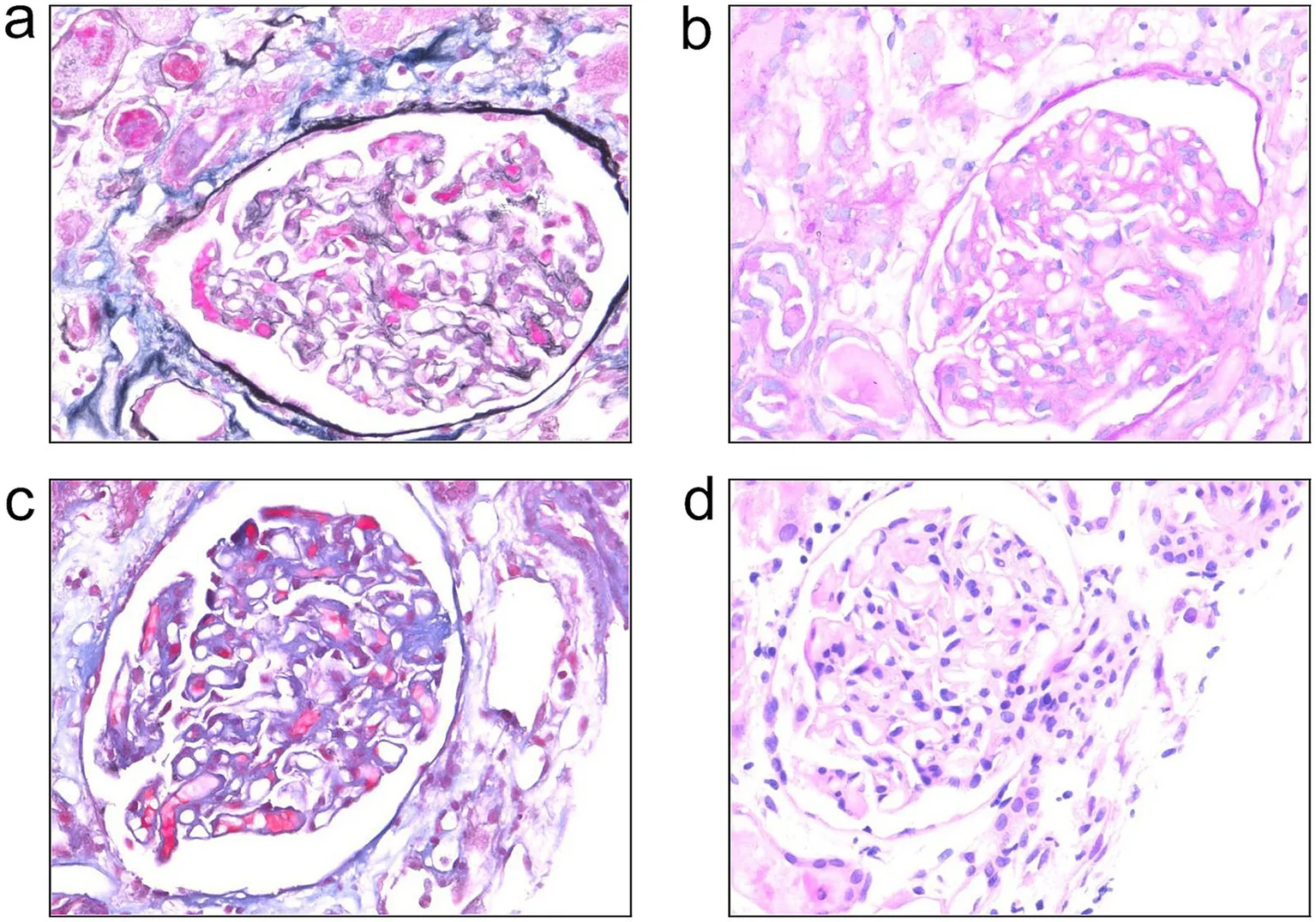
Kidney biopsy findings: **(a)** Massive thrombi in the glomerular capillary lumens, and the glomerular basement membrane showing a double-track sign (PASM, magnification × 40). **(b)** Polymorphonuclear leukocyte within the capillary loops, vascular endothelial swelling with narrowing of the capillary lumen, and GBM showing a double-track sign (PAS, magnification × 40). **(c)** Thrombi in the glomerular capillary cavity, and narrowing of the glomerular capillary lumen (Masson, magnification × 40). **(d)** Transplant glomerulitis with narrowing of the capillary lumen, and significant thickening of the glomerular basement membrane (HE, magnification × 40).

On September 15, 2024, the patient presented with acute-onset right lower abdominal pain and swelling accompanied by reduced 24-h urine output (1,200 mL/24 h). Clinical examination revealed significant hypertension (166/103 mmHg) and bilateral pitting edema of the lower limbs, necessitating hospital readmission for graft dysfunction evaluation. Laboratory investigations revealed markedly elevated fasting glucose (25 mmol/L), acute kidney injury (serum creatinine 408 μmol/L, peak 711 μmol/L), severe thrombocytopenia (75 × 10^9^/L, nadir 7 × 10^9^/L), mild proteinuria (0.96 g/24 h), and substantially elevated LDH (1,070 U/L, peak 1,220 U/L). Comprehensive serological testing revealed negative results for HLA/non-HLA antibodies, BK virus, and anti-phospholipase A2 receptor antibodies. Lymphocyte subset analysis indicated mildly elevated postoperative T-cell (154/μL) and B-cell (186/μL) counts. Renal transplant Doppler ultrasound demonstrated increased intrarenal arterial resistance with a prolonged acceleration time. The diagnostic workup showed preserved ADAMTS13 activity (69.4%) and excluded Shiga toxin-producing *Escherichia coli* infection. Complement studies revealed elevated C5b-9 membrane attack complex levels (300 ng/mL), while allograft biopsy demonstrated glomerular basement membrane duplication, transplant glomerulitis, and endothelial swelling, confirming thrombotic microangiopathy (Figures 1a–d). Renal transplant biopsy revealed cortical, corticomedullary, and medullary tissue. Some capillary loops showed congestion and dilation, with extensive thrombi within the glomerular capillary lumens. The glomerular basement membrane exhibited a double-contour sign (cg1b). Tubular epithelial cells demonstrated vacuolar and granular degeneration, focal loss of brush border, flat segments with exposed basement membranes, and small foci of atrophy (ct1). Occasional tubulitis (t1) was observed, with a few protein casts and sloughed epithelial cells in the tubules. Interstitial infiltration consisted of scattered lymphocytes, monocytes, and occasional neutrophils (i10), accompanied by fibrosis (ci1). Mild thickening of the small arterial walls was noted without definite arteriitis (v0) or peritubular capillaritis (ptc0). Immunohistochemistry revealed C4d (C4d0), CD3 (T-cell+), CD4 (partial T-cell+), and CD8 (partial T-cell+). After the diagnosis of TMA, plasma exchange was not performed because of the presence of a closed arteriovenous fistula (AVF) and patient refusal of temporary central venous catheterization. Following treatment with eculizumab (900 mg weekly for the first four weeks, then 1,200 mg weekly from week five to twelve), the clinical symptoms significantly improved. Due to the patient’s prior history of allergy to the influenza vaccine, meningococcal vaccination declined; thus, prophylactic amoxicillin was administered to prevent meningococcal infection. Three months after therapy initiation, the patient’s clinical condition markedly improved.

**Figure 2:**
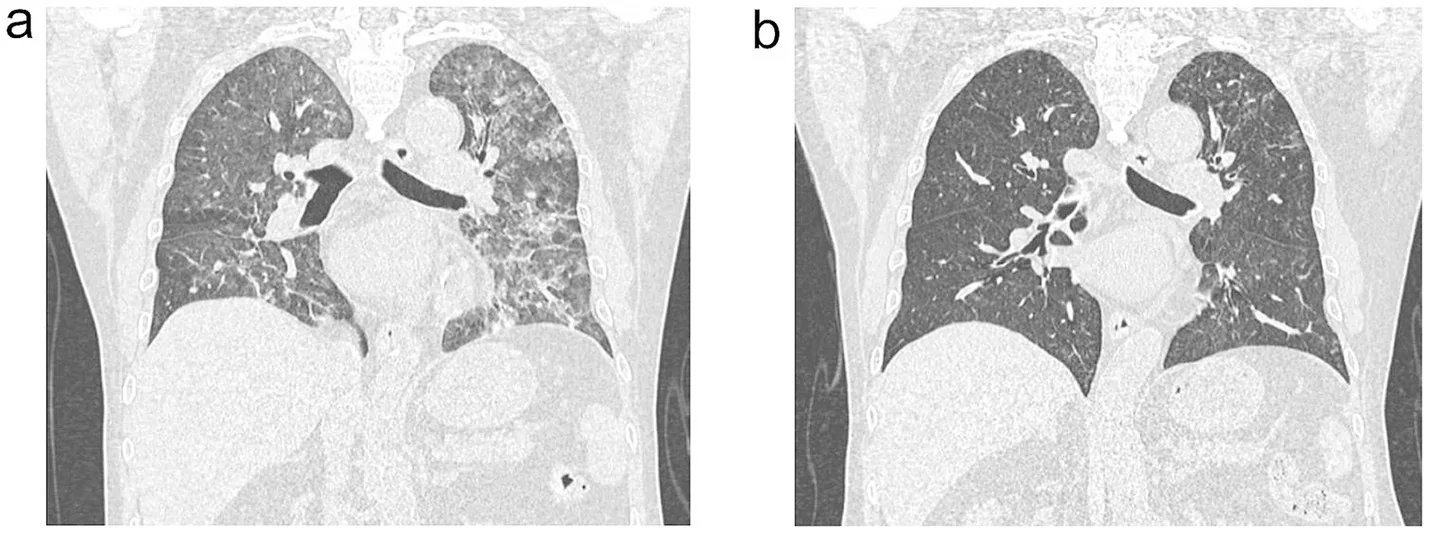
Chest CT scans: **(a)** Bilateral pneumonia (as to radiological findings, the most common patterns were multiple patchy, linear shadows and ground-glass opacity). **(b)** The reexamination of the chest CT showed that the absorption of lung exudates was better than before.

During the treatment course, two opportunistic infections occurred between December 3, 2024, and January 5, 2025. The patient was first hospitalized on December 3, 2024, for perianal herpes, which resolved following a 14th-day course of acyclovir (500 mg three times daily). One week post-discharge, he presented to the emergency department with chest discomfort and was readmitted after a chest CT demonstrated bilateral pneumonia with a concomitant fever of 38.5 °C (Figure 2a). Metagenomic next-generation sequencing (mNGS) of bronchoalveolar lavage fluid identified Pneumocystis jirovecii and human cytomegalovirus, confirming concurrent Pneumocystis pneumonia and Cytomegalovirus (CMV) pneumonitis. The infection was resolved with a combination of antimicrobial therapy (Figure 2b), including piperacillin-tazobactam (4.5 g three times daily for two weeks), caspofungin (50 mg daily for 15 days), trimethoprim-sulfamethoxazole (0.96 g twice daily for 15 days), ganciclovir (0.25 g twice daily for two weeks), and IVIG (10 g daily for ten days), with subsequent discharge following clinical recovery.

**Figure 3:**
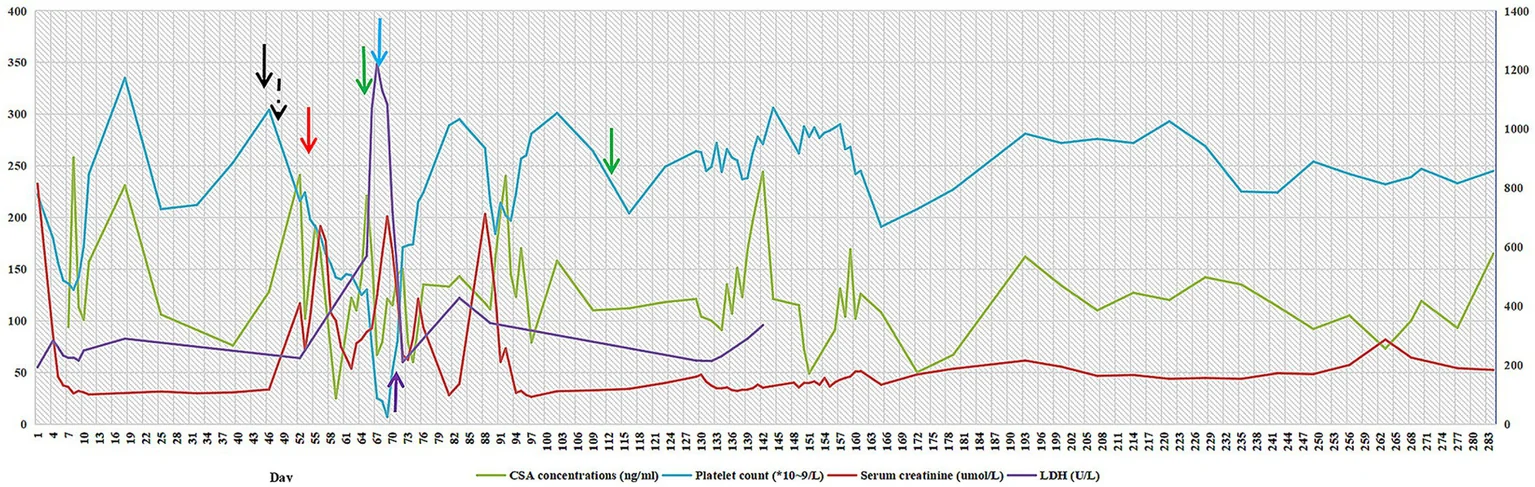
Laboratory test results: Trend of CSA concentrations (ng/ml), platelet count (×10^9^/L), serum creatinine (umol/L), and LDH (U/L) after kidney transplantation; the left-side vertical axis shows CSA concentrations and platelet count, while the right-side vertical axis shows serum creatinine and LDH. The black full arrow indicates presentation at the Emergency Department (day 46). The dashed arrow indicates steroid pulse therapy (days 49, 50, and 51). The red full arrow indicates administration of ATG (days 54, 55, 56, and 57). The blue full arrow indicates an allograft biopsy (day 69). The green full arrow indicates initiation (day 66) and discontinuation (day 114) of eculizumab. The purple full arrow indicates the last transfusion of platelets (day 71).

Nine months after eculizumab discontinuation, the patient remained free from TMA relapse, thrombocytopenia recurrence, or dialysis-requiring acute kidney injury (Figure 3). The graft function was good. The serum creatinine level fluctuated slightly due to changes in the blood concentration of CSA, but remained stable at approximately 180 μmol/L. The last re-examination showed a serum creatinine level of 149 μmol/L, C5b-9 membrane attack complex concentration of 220 ng/mL, and proteinuria of 0.36 g/day.

## 3 Discussion and conclusion

To the best of our knowledge, previous studies have identified that TMA can be triggered by immunosuppressive drugs and ABMR. However, ATCR-associated TMA has rarely been reported, particularly among patients with type 2 diabetes mellitus, and this is the first such case report in China. Here, we report the first successful use of eculizumab monotherapy (without PE) at our institution for *de novo* TMA development in the context of ATCR, type 2 diabetes mellitus, and post-transplant immunosuppression. Both hyperglycemia and ATCR contribute to renal microcirculatory impairment through complement-dependent and independent pathways, including endothelial injury and impaired repair mechanisms mediated by alternative complement pathway dysregulation. Importantly, CSA nephrotoxicity contributes to allograft dysfunction not only through direct tubular injury, but also by promoting microthrombosis via altered nitric oxide, prostacyclin, thromboxane, thrombomodulin, endothelin, and von Willebrand factor homeostasis (7). Although we clearly identified elevated cyclosporine levels as a contributing factor to TMA, this patient had a history of type 2 diabetes. If tacrolimus was used as the primary immunosuppressive agent, the side effects of hyperglycemia would be significantly more pronounced than those of cyclosporine. Therefore, after careful consideration, we continued with a cyclosporine-based treatment regimen and closely monitored the drug concentration. In this case, the patient did not undergo preoperative kidney biopsy to confirm the underlying renal disease. Although the patient reported negative results from genetic testing for TMA-related genes performed at another hospital, despite our repeated communication attempts and lack of access to the written report, the patient refused to undergo further genetic testing. This represents a significant limitation, as undetected complement gene variants can substantially influence TMA susceptibility and recurrence risk.

Renal allograft biopsy remains the gold standard for definitive diagnosis and is generally well tolerated; however, identified risk factors for post-procedural bleeding complications (including platelet counts below 50 × 10^9^/L) enable clinicians to optimize patient selection and implement appropriate monitoring strategies (e.g., platelet transfusion and biopsy timing optimization) (8). In this case, the biopsy was performed when the platelet count was 22 × 10^9^/L as part of routine clinical practice, with no subsequent hemorrhage, hematoma, or surgical complications observed. Nevertheless, to maximize patient safety and safeguard clinical outcomes, we strongly recommend maintaining platelet counts above 50 × 10^9^/L prior to biopsy. For counts below this threshold, a comprehensive clinical assessment is essential, with prophylactic platelet transfusion advised when necessary to mitigate bleeding risk.

Both laboratory markers and renal transplant Doppler ultrasound demonstrated ATCR prior to TMA diagnosis. The patient subsequently received intensified immunosuppression with ATG and IVIG because of an inadequate response to the initial methylprednisolone pulse therapy. This combination therapy proved effective, with SCr rapidly decreasing to 200 μmol/L (Figure 3). However, serial monitoring revealed subsequent SCr elevation beginning on October 2, 2024, which correlated with elevated CSA concentrations, increased LDH levels, and thrombocytopenia.

TA-TMA should be considered in cases of unexplained renal impairment after excluding more common etiologies, such as calcineurin inhibitor nephrotoxicity, ABMR, and BK virus-associated nephropathy. The patient’s transplant kidney biopsy showed CD4d negativity and negative HLA antibody screening; based on these findings, we ruled out antibody-mediated rejection-related TMA. The patient’s postoperative cyclosporine trough levels were consistently within the normal range, making CNI toxicity an unlikely primary etiological factor. BK virus testing was negative, as were tests for Shiga toxin-producing *Escherichia coli* and other bacterial infections, thus excluding infection-related TMA. Diabetic microangiopathy is a long-term complication of diabetes; however, given the short duration since transplantation and well-controlled blood glucose levels post-surgery, diabetic microangiopathy-related TMA was not considered, and the diagnosis was ultimately confirmed through quantification of complement C5b-9 membrane attack complex and allograft biopsy, although the latter carries a significant bleeding risk in thrombocytopenic patients (4).

Before eculizumab became available, plasma-based therapies were the primary treatments for TMA. However, this patient did not undergo PE for several reasons. First, peripheral vascular access was compromised due to AVF occlusion on September 25, 2024, and the patient declined venous catheter placement given its higher postoperative complication and mortality rates than fistulas. Second, laboratory investigations confirmed that this was an anti-CFH antibody-negative TMA case without DSA, a plasmapheresis-resistant form that is unlikely to respond to PE (administered every other day for 11 days) or rituximab (limited to single-dose administration). Third, plasma therapy demonstrates reduced efficacy in *de novo* or recurrent post-transplant TMA with complement gene expression deficiency (9). Fourth, therapeutic plasmapheresis shows limited effectiveness in cases without quantitative ADAMTS13 defects or ABMR (10–12). Finally, continuous monitoring of renal tubular function and urine output during 24-h urine output withinTMA treatment revealed persistently normal parameters (urine specific gravity 1.010; the normal range), obviating the need for venous catheter placement and high-efficiency hemodialysis based on renal function assessments. In cases demonstrating resistance to conventional therapies with contraindications to PE, eculizumab represents a potential graft-preserving and life-saving therapeutic alternative.

Eculizumab, a monoclonal antibody that specifically binds to complement factor C5, effectively inhibits terminal complement pathway activation and prevents endothelial cell injury (10). This treatment has demonstrated efficacy in patients with TA-TMA, severe ABMR, and recurrent TMA (3). In contrast to the previous cases, the current patient achieved complete clinical remission after eculizumab monotherapy. Several critical factors influence therapeutic decision making and outcomes in these cases, including the efficacy and safety profile of rescue therapies, long-term risks associated with eculizumab (particularly infectious complications), and significant treatment costs. Currently, limited clinical data exist regarding eculizumab use for TA-TMA management, and no consensus has been established to guide the treatment duration. For this patient, the treatment protocol followed the guidelines developed by a Dutch national working group, which recommends reserving eculizumab for rescue therapy in confirmed cases of recurrent or *de novo* TMA rather than prophylactic use in kidney transplant recipients, with consideration of discontinuation after three months of therapy (6). The SETS-AHUS trial indicates that, provided regular monitoring of relevant indicators is conducted, discontinuing eculizumab does not carry a higher risk of adverse effects compared to continuous treatment, and due to the high cost of eculizumab, it can reduce treatment expenses (13). At 9 months following the final dose of eculizumab as rescue therapy for TA-TMA, our clinical observations confirm maintained allograft function with no disease recurrence. Eculizumab has a favorable safety profile, with nasopharyngitis, headache, back pain, and nausea being the most frequently reported adverse effects in clinical trials (9, 11). However, by inhibiting the terminal complement pathway, this treatment increases the susceptibility to meningococcal and other encapsulated bacterial infections (12). Current US guidelines recommend meningococcal vaccination for eculizumab recipients, but do not mandate long-term antibiotic prophylaxis (3, 14). In this case, the patient refused to receive the quadrivalent meningococcal polysaccharide vaccine due to a previous allergic reaction to the influenza vaccine and chose to use antibiotics for prophylaxis. Following nine months of eculizumab discontinuation, the patient remained free from TMA relapse with excellent graft function and no proteinuria.

During the treatment course, the patient developed severe herpes infection and concurrent Pneumocystis jirovecii pneumonia (PJP) with cytomegalovirus (CMV) pneumonitis. This clinical outcome highlights the significant infection risk associated with intensive combined immunosuppression (incorporating ATG, corticosteroids, CNI, and MMF) coupled with complement pathway inhibition (eculizumab). A key limitation of this case is the concurrent administration of SPT, ATG, and IVIG—all potent therapies for acute cellular rejection, particularly T cell-mediated rejection. Given that rejection episodes may trigger TMA, the observed therapeutic response could potentially reflect anti-rejection treatment efficacy rather than eculizumab-specific effects. This underscores the need for future large-scale clinical studies to definitively establish the therapeutic role of eculizumab in TMA management.

In summary, the early initiation of comprehensive treatment with eculizumab may benefit patients with TMA refractory to PE. This case demonstrates the unique finding that eculizumab monotherapy without prior plasmapheresis can achieve rapid improvement in renal function. We recommend prompt consideration of eculizumab-based regimens for transplant recipients with complement-mediated TMA, as evidenced by both biochemical and histopathological features.

## Data Availability

The original contributions presented in the study are included in the article/supplementary material, further inquiries can be directed to the corresponding authors.

## References

[1] Pan T Qi J Tang Y Yao Y Chen J Wang H . N-acetylcysteine as prophylactic therapy for transplantation-associated thrombotic microangiopathy: a randomized, placebo-controlled trial. Transplant Cell Ther. (2022) 28:764.e1–7. doi: 10.1016/j.jtct.2022.07.029, 35940529

[2] Merola J Yoo PS Schaub J Smith JD Rodriguez-Davalos MI Tichy E . Belatacept and eculizumab for treatment of calcineurin inhibitor-induced thrombotic microangiopathy after kidney transplantation: case report. Transplant Proc. (2016) 48:3106–8. doi: 10.1016/j.transproceed.2016.04.005, 27932157

[3] Ikeda T Okumi M Unagami K Kanzawa T Sawada A Kawanishi K . Two cases of kidney transplantation-associated thrombotic microangiopathy successfully treated with eculizumab. Nephrology. (2016) 21:35–40. doi: 10.1111/nep.12768, 26970541

[4] Godara A Migliozzi DR Pilichowska M Goyal N Varga C Gordon CE. Use of eculizumab in transplant-associated thrombotic microangiopathy in a patient with polycystic kidney disease immediately post-kidney transplant: a case report. Kidney Med. (2020) 2:652–6. doi: 10.1016/j.xkme.2020.06.007, 33089142 PMC7568057

[5] Vanikar A Kanodia K Suthar K Nigam L Patel R Thakkar U . Thrombotic microangiopathy in a renal allograft: single-center five-year experience. Saudi J Kidney Dis Transpl. (2020) 31:1331–43. doi: 10.4103/1319-2442.308342, 33565445

[6] Duineveld C Bouwmeester RN Wijnsma KL Bemelman FJ Van Der Heijden JW Berger SP . Eculizumab rescue therapy in patients with recurrent atypical hemolytic uremic syndrome after kidney transplantation. Kidney Int Rep. (2023) 8:715–26. doi: 10.1016/j.ekir.2023.01.016 37069997 PMC10105043

[7] Young JA Pallas CR Knovich MA. Transplant-associated thrombotic microangiopathy: theoretical considerations and a practical approach to an unrefined diagnosis. Bone Marrow Transplant. (2021) 56:1805–17. doi: 10.1038/s41409-021-01283-0, 33875812 PMC8338557

[8] Lim CY Khay SL. Bleeding complications after percutaneous kidney biopsies—nationwide experience from Brunei Darussalam. World J Nephrol. (2023) 12:147–58. doi: 10.5527/wjn.v12.i5.147, 38230299 PMC10789084

[9] Hillmen P Brodsky RA Röth A Nakamura R Hill A Maciejewski J . The complement inhibitor eculizumab in paroxysmal nocturnal hemoglobinuria. N Engl J Med. (2006) 355:1233–43. doi: 10.1056/NEJMoa061648, 16990386

[10] Guzzo G Kissling S Pantaleo G Pascual M Sadallah S Teta D. Complement activation and blockade in massive post-partum haemorrhage, thrombotic microangiopathy and acute kidney injury: a case report. BMC Nephrol. (2021) 22:252. doi: 10.1186/s12882-021-02456-1, 34229609 PMC8259140

[11] Rathbone J Kaltenthaler E Richards A Tappenden P Bessey A Cantrell A. A systematic review of eculizumab for atypical haemolytic uraemic syndrome (aHUS). BMJ Open. (2013) 3:e003573. doi: 10.1136/bmjopen-2013-003573, 24189082 PMC3822313

[12] Tanimoto T Kusumi E Hosoda K Kouno K Hamaki T Kami M. Concerns about unapproved meningococcal vaccination for eculizumab therapy in Japan. Orphanet J Rare Dis. (2014) 9:48. doi: 10.1186/1750-1172-9-48, 24716834 PMC3990028

[13] Mirioglu S Morelle J Efe O Hurdogan O Dirim AB Kumru G . Thrombotic microangiopathy after kidney transplantation: diagnosis and management strategies. Clin Kidney J. (2026) 19:sfag018. doi: 10.1093/ckj/sfag018, 41822056 PMC12976228

[14] Bozio CH Isenhour C McNamara LA. Characteristics of and meningococcal disease prevention strategies for commercially insured persons receiving eculizumab in the United States. PLoS One. (2020) 15:e0241989. doi: 10.1371/journal.pone.0241989, 33180804 PMC7660549

